# Equity, diversity and inclusion in simulation-based education: constructing a developmental framework for medical educators

**DOI:** 10.1186/s41077-024-00292-5

**Published:** 2024-05-16

**Authors:** Jennifer Mutch, Shauna Golden, Eve Purdy, Chloe Hui Xin Chang, Nathan Oliver, Victoria Ruth Tallentire

**Affiliations:** 1 Medical Education Directorate, NHS Lothian, Waverley Gate, 2–4 Waterloo Place, Edinburgh, EH1 3EG Scotland; 2 NHS Fife, Victoria Hospital, Hayfield Road, Kirkcaldy, KY2 5AH Scotland; 3 Gold Coast Hospital and Health Services, Southport, QLD Australia; 4 Faculty of Health Sciences & Medicine, Bond University, Gold Coast, QLD Australia; 5 University Hospital Wishaw, Netherton Street, Wishaw, ML2 0DP Scotland; 6 University of Canberra, 11 Kirinari Street, Canberra, ACT, 2617 Australia

**Keywords:** Healthcare, Simulation, Simulation-based education, Equity, diversity and inclusion, Faculty development

## Abstract

**Background:**

Themes of equity, diversity and inclusion (EDI) arise commonly within healthcare simulation. Though faculty development guidance and standards include increasing reference to EDI, information on how faculty might develop in this area is lacking. With increasingly formal expectations being placed on simulation educators to adhere to EDI principles, we require a better understanding of the developmental needs of educators and clear guidance so that teams can work towards these expectations. Our study had two aims: Firstly, to explore the extent to which an existing competency framework for medical teachers to teach ethnic and cultural diversity is relevant for simulation educator competency in EDI, and secondly, informed by the data gathered, to construct a modified competency framework in EDI for simulation educators.

**Methods:**

We engaged our participants (10 simulation faculty) in a 5-month period of enhanced consideration of EDI, using the SIM-EDI tool to support faculty debriefing conversations focussed on EDI within a pre-existing programme of simulation. We interviewed participants individually at two timepoints and analysed transcript data using template analysis. We employed an existing competency framework for medical teachers as the initial coding framework. Competencies were amended for the simulation context, modified based on the data, and new themes were added inductively, to develop a new developmental framework for simulation educators.

**Results:**

Interview data supported the relevance of the existing competency framework to simulation. Modifications made to the framework included the incorporation of two inductively coded themes (‘team reflection on EDI’ and ‘collaboration’), as well as more minor amendments to better suit the healthcare simulation context. The resultant Developmental Framework for Simulation Educators in EDI outlines 10 developmental areas we feel are required to incorporate consideration of EDI into simulation programmes during the design, delivery and debriefing phases. We propose that the framework acts as a basis for simulation faculty development in EDI.

**Conclusions:**

Simulation faculty development in EDI is important and increasingly called for by advisory bodies. We present a Developmental Framework for Simulation Educators in EDI informed by qualitative data. We encourage simulation teams to incorporate this framework into faculty development programmes and report on their experiences.

**Supplementary Information:**

The online version contains supplementary material available at 10.1186/s41077-024-00292-5.

## Introduction

Themes of equity, diversity and inclusion (EDI) arise within healthcare simulation independent of scenario design and regardless of whether we as faculty feel equipped to address them. Though faculty development guidance and standards increasingly reference EDI, we see this as relatively broad brushstrokes. There is a dearth of information on how simulation faculty can develop in this area [1–4]. With increasingly formal expectations being placed on simulation educators to adhere to EDI principles, we must obtain a better understanding of the developmental needs of educators in this area. The gap in both clarity and specificity in guidance risks creating confusion amongst simulation practitioners and holds a latent risk of unintentional harm. This paper seeks to address these issues to enable educators to rise to the challenge of incorporating EDI in simulation, better meeting the needs of learners and their future patients.

### Equity, diversity and inclusion (EDI)

Equity, diversity and inclusion are terms that refer to different aspects of interactions between people and within groups [5]. It is well established that those who identify as members of marginalised and underrepresented groups, and those exposed to social and economic disadvantage, experience barriers, both systemic and structural, which prevent them from receiving safe, effective and equitable care [6–8].

NHS Education for Scotland provides this definition of EDI in their Advancing Equity in Medical Education resources [9]: equity — creating a fairer society where everyone has the opportunity to fulfil their potential; diversity — recognising and valuing difference in its broadest sense; and inclusion — celebrating individual differences to ensure everyone feels welcome and accepted. Different conceptions and definitions exist; for the purposes of this study, we have used the definitions provided above. We acknowledge that other combinations of related concepts are encountered across academic fields (e.g. equ*al*ity, diversity and inclusion; justice, equity, diversity and inclusion (JEDI)); however, ‘EDI’ is the prominent lens through which academic discourse in simulation-based education (SBE) has been framed and the lens through which we consider simulation faculty development in this study.

### EDI in simulation-based education

#### The reason

Over recent years, increasing light has been shone on EDI within SBE literature. This is in relation to simulation with specific EDI learning objectives and the consideration of EDI in all SBE programmes in healthcare [5, 6, 10–13]. SBE in healthcare has been described as a time of ‘cultural compression’, where ideologies about the healthcare professions can be reinforced, and values and beliefs can be transmitted to learners with intensity [14]. When EDI themes are explicitly incorporated into simulation education, numerous positive impacts on participants are reported. These include the following: increase in self-awareness, enhanced communication, enhanced insight and knowledge, strengthening in EDI-related self-efficacy and increased EDI-related competence and skills [10]. This concept of cultural compression as it relates to simulation also supports the need for educators delivering SBE without the explicit incorporation of EDI themes to have awareness, knowledge and skills in this area to avoid causing or perpetuating harm. The growing attention to EDI within simulation has been backed by increasing calls within professional standards and codes of conduct for consideration of EDI [1–4].

The Association for Simulated Practice in Healthcare (ASPiH) includes equity, diversity and inclusion as a core value in their ‘Standards guiding simulation-based practice in health and care’ and calls for continuing professional development in EDI to be part of all simulation faculty development programmes [1]. They outline that training should ‘as a minimum’ result in faculty who can promote EDI within the design and delivery of simulation, prevent harm arising from ‘tokenism, misrepresentation, stereotyping or microaggressions’ and highlight the importance of diversity in improving the learning environment [1]. Honouring diversity and fostering inclusion are part of the values within the Healthcare Simulationist Code of Ethics [3]. The Academy of Medical Educators includes ‘Demonstrates respect for others’ as a core value of medical educators, further outlining the expectation that educators will ensure ‘equality of opportunity for patients, students, trainees, staff and colleagues’ and ‘actively promote[s] and respect[s] diversity in discharging their educational responsibilities’ [4].

#### The challenge

Many simulation teams recognise the need to incorporate EDI into their programmes and are motivated to make improvements. Initiating this shift however presents a significant challenge. Studies of simulation educators report a perceived lack of cultural knowledge and confidence, a lack of clarity on where to focus their efforts and a lack of understanding of how to meaningfully address EDI in simulation without causing harm [13, 15, 16].

Purdy et al. highlighted this gap between academia and action [13]. They argued that development opportunities for faculty are crucial to empower them to incorporate EDI meaningfully and safely [13, 17]. Despite the increasing discussion of a need for the incorporation of EDI within simulation, and the recognition that we must upskill our educators to address EDI, there are currently no frameworks that outline EDI competencies for simulation faculty development.

## Methods

### Aims

The aims of the study were twofold.

1. To explore the extent to which an existing competency framework for medical teachers to teach ethnic and cultural diversity is relevant for simulation educator competency in EDI.
2. To construct a modified competency framework in EDI for simulation educators.

### Study design

In this constructivist study, participants (simulation faculty) were engaged in a 5-month period of enhanced consideration of EDI. This involved using the SIM-EDI tool [13] to support faculty debriefing conversations focussed on EDI within a pre-existing programme of simulation. Study participants were interviewed individually at two timepoints to explore their understanding of EDI concepts, their experiences and perceptions of EDI within simulation and their views on faculty development in EDI. Interview questions were designed to draw out data of relevance to Hordijk et al.’s teaching competency framework [18]. Analysis was completed using template analysis, employing an amended version of Hordijk et al.’s framework as the initial coding template. Competencies were modified by the data, and new themes were developed inductively to construct a new competency framework in EDI for simulation educators.

### The SIM-EDI tool

SIM-EDI [13] is a tool designed to prompt and guide reflexive conversations amongst simulation faculty following the delivery of simulation sessions. It guides simulation teams to consider EDI in the design, delivery and debriefing of simulation and prompts discussion of missed opportunities, harms, potential biases and action items as they relate to the simulation session just delivered. Participants were introduced to the tool by J. M. in an information session in December 2022 and were supported directly by J. M. and S. G. during early uses of the tool. The EDI debriefing conversations involved faculty only (no learners) and took place following simulation sessions delivered as part of a regular programme of education running within the department of medical education. SIM-EDI is considered a methodological tool [19] in this study. Use of the tool enhanced awareness amongst participants of EDI within simulation, familiarised them with relevant concepts and provided the vocabulary to be able to identify and describe experiences and developmental needs as they relate to EDI. Thus, the use of SIM-EDI supported the collection of meaningful data in the second set of interviews.

### Setting

The study was conducted in NHS Lothian, a National Health Service (NHS) Board in the Southeast of Scotland. The simulation team sits within the Medical Education Directorate and provides a variety of simulation programmes for a range of healthcare professionals.

### Simulation

The study involved use of SIM-EDI within the pre-existing core simulation programme in NHS Lothian. This programme consists of a series of simulation sessions delivered for doctors in their foundation years, the first 2 years of postgraduate medical training in the UK. A range of topics are covered within the programme including acute medical assessment, psychiatric assessment and management and challenging communication scenarios.

### Interviews

Semi-structured interviews were conducted in accordance with interview schedules developed by the research team based on Hordijk et al.’s teaching competency framework (Additional file 1). All interviews were conducted via video call using Microsoft Teams. Interviews were transcribed verbatim directly through Microsoft Teams and checked for accuracy by the interviewer, with clarifications made directly with the participant where required. Initial interviews in January 2023 were conducted by J. M. who was a medical education fellow known to the participants. Interviews at the second time point, April 2023, were conducted by C.H.X.C., at the time a clinical teaching fellow working in a different Health Board and not known to the participants. The aim of this design (with interviews at two time points) was to try and capture perceived developmental needs or established competencies that faculty ‘arrive with’, developed through professional and personal experiences, and subsequently to capture those that are recognised and/or developed during the early stages of using a reflexive tool. The choice of interviewer was made with the aim of promoting open and uninhibited discussion in the second set of interviews, where reflections on the process of enhanced consideration of EDI in the team were sought (a process which J. M. and S. G. had supported). Individual interviews were chosen to allow exploration of sometimes challenging and sensitive topics [20] and to support open reflection on individual values and beliefs.

### Analysis

Hordijk et al.’s framework for medical teachers’ competencies to teach ethnic and cultural diversity [18] (hereafter referred to as the ‘original framework’) was used as the initial coding framework. This framework, developed by a Delphi method, was the only faculty development framework relating to EDI that we identified in the literature. We considered the original framework as a robustly developed set of educational competencies on which to build our study. The 10 competencies were used as predefined themes, which were amended for our context prior to analysis and modified further during analysis based on the emerging data creating an ‘amended framework’. This is in line with a template analysis approach [21]. New themes emerging from the data were coded inductively [21]. Details of the amended framework, with timing and justification of amendments, can be seen in Additional file 2. J. M. and S. G. coded each transcript independently. Discrepancies in coding to the amended framework and emerging themes were discussed and agreed before recoding of all transcripts in accordance with the newly developed definitions and shared understanding. E. P. and V. T. each independently coded one randomly selected transcript from the January interviews and one from the April interviews. Any discrepancies in coding were discussed with J. M. and S. G. before final coding was agreed. Amendments to the original framework based on the data, in addition to new themes emerging from the data, formed the basis of a new ‘Developmental Framework for Simulation Educators in EDI’. The new framework developed in this constructivist study is J. M. and S. G.’s conceptualisation of the data produced through interactions between J. M., S. G., co-researchers and participants. The concept of an objective reality is rejected in this work.

### Ethics

Ethical approval was received from the University of Edinburgh Medical Education Ethics Committee (reference number: 2022/37). Written consent was obtained from all participants for audio and video data collection and publication of anonymised results. All participants were free to leave the study at any time.

## Results

All 10 members of simulation faculty involved in the delivery of the core simulation programme at the time of the study consented to take part. Seven were medical education fellows, and three were simulation technicians. Some of the participants had additional experience as faculty in other simulation programmes. The participants had a range of simulation experience and professional backgrounds, though relatively limited diversity in age, LGBTQ, religion and ethnicity. Demographic characteristics of participants can be seen in Table 1.

**Table 1:** Demographic characteristics of participants

Characteristic	Category	Number of participants
Age	20 s	1
	30 s	9
Gender	Female	5
	Male	5
	Other	0
Ethnicity	White Scottish	7
	White Irish	1
	Mixed or multiple ethnic groups: White — Other British and Asian (Sri Lankan)	1
	Other: Jewish	1
Job role/clinical specialty	Simulation technician	3
	Geriatric medicine	1
	Oncology	1
	Surgery	1
	Emergency medicine	1
	Paediatrics	1
	Palliative care	1
	Public health	1

Participants used the SIM-EDI tool to guide 23 EDI debriefing conversations between January and April 2023. Each EDI debriefing conversation involved between two and five participants. Nine of the 10 participants used the SIM-EDI tool on more than one occasion. Interviews were conducted with all participants (P1 to P10) at two timepoints (I1 and I2); in January, interviews lasted between 18 and 37 min (mean 27 min) and in April between 12 and 26 min (mean 17 min).

### Relevance of an existing teaching competency framework for medical teachers in ethnic and cultural diversity to simulation educator competency in EDI

The competencies in the amended framework are presented in Table 2 alongside findings from the interview data and illustrative quotes.

**Table 2:** Amended framework competencies, findings from interview data and illustrative quotes

Competency	Findings	Illustrative quotes
1. Ability to reflect on own values and beliefs	Participants (P9, P4) described how the ability (or lack thereof) to reflect on their own values and beliefs could impact on simulation through reinforcement of stereotypes within scenarios or debriefings. Participants (P7, P8) highlighted that their own values changed over the study period, through heightened awareness of the relevant issues, altered understanding of the concept of EDI and the challenging of personal perceptions.	‘It's really easy to stereotype and we do it all the time in medicine and I think when you actually start recognising it and challenging it, I think people start to see that too’. [P4, I2] ‘I certainly have considered what my own unconscious biases are, and continue to reflect on that, and think about what you need to do to mitigate against them. So, I think that's a good starting point’. [P7, I1] ‘I guess equality and equity is another angle that I consider a lot more now than I used to. It was always, culture, race, sort of stuff, but power imbalance playing a role in how people are treated is something that I'm just a lot more aware of now’. [P8, I2]
2. Ability to communicate about individuals from ethnic, social, cultural and professional groups in a nondiscriminatory, non-stereotyping way	Participants (P1, P7) were concerned with having the correct language to be able to communicate confidently in a nondiscriminatory and non-stereotyping way when facilitating simulation. Specific examples reflected on included gendered language and use of correct pronouns (P2). One participant (P5) highlighted how the focus on language may have a negative impact on the simulation educator’s own psychological safety, finding it paralysing and distracting within a simulation session.	‘I don't quite know the words to use, so that could hold people back because they're like, “I don't even know what I can say anymore without offending somebody”. I'm just so aware of that and how damaging that can be for people’. [P1, I1] ‘You know, I think gender and race is one thing, but sexual orientation… I think just knowing how to communicate sensitively, for non-binary and transgender people, how you don't get caught up with the wrong words’. [P2, I1] ‘I guess, in a weird way, it maybe affects my psychological safety because the whole time I'm thinking “ohh gosh use the right terminology, don't say the wrong thing to offend” and, yeah, I'm just so focused on that and I wonder if that may impact the participants experience because they would know that’. [P5, I1]
3. Empathy (understanding and compassion) for all patients and people, being mindful of ethnicity, race or nationality, sex, gender, cultural background, neurodiversity, socioeconomic status, body habitus	One participant (P3) described learners demonstrating a lack of empathy for patients who attended hospital frequently due to issues relating to substance misuse. Though they recognised empathy in themselves for patients in this situation, they felt unable to explore the issue further with learners. Another (P9) reflected on lack of empathy for patients having the potential to lead to the conclusion that a person’s background is the reason for the difficulties that they present with. Reflections (P1, P7, P8) extended to how educators might empathetically represent people within simulation from a place of understanding and compassion without stereotyping, considering the roles of collaboration and co-creation as a way of achieving this.	‘One of the students talked about how some patients they could relate to and see as peers and other patients were kind of “frequent flyers” to ED, so they were kind of churning them out quite quickly, wouldn't give them that much time, time to build a rapport. And I think that's a real harm because it's one I've seen in my clinical practice of different care for patients depending on their class background or socioeconomic status, or if they've substance history or substance misuse. And I think that’s one that's very prevalent in clinical care and is shown to affect clinical outcomes, I think that's one I would have preferred to have challenged as a debriefer or like encourage reflection, and I didn't really feel equipped to unpick that. So I sort of brushed over it I think maybe. Yeah, that was really hard’. [P3, I1] ‘You may find that the vast majority of the sim team maybe share similar backgrounds… but actually to go and understand the mindset and experience of those in minority groups. That's going to give us that experience to see what it looks like in real life and how we can actually stop that happening, for them to have these bad experiences. So, I think the approach should be to recognise the experiences of these groups and think, right when we were doing our sims, how can we be mindful of that?’ [P1, I1] ‘You know for example, our psych programme, have we included any of the voices of people, psychiatry patients, what would they want doctors to know about common psychiatric conditions? What would they want them to know about their experience of being a relative of an agitated patient with delirium? You know we have assumed that this is what the doctors should know, but really we're acting in the care of our patients, so why haven't we asked them what's in their interest? How could we include them?’ [P7, I1]
4. Awareness of intersectionality (different interrelated dimensions of one person/patient, e.g. culture, social class, gender, disability, religion, sexual orientation)	No excerpts were coded to this competency.	No relevant quotes
5. Awareness of own ethnic and (sub)cultural background/standards **and those of the team/staff delivering simulation education**	Participants (P1, P4, P8) described their perceptions of how their personal backgrounds might limit or influence their abilities in relation to the incorporation of EDI into simulation. They highlighted the importance of awareness of, and reflection on, the backgrounds of the simulation team as a group. Concerns were voiced (P5, P7, P8) regarding lack of diversity within the team limiting the team’s ability to bring diversity into scenarios and the subsequent potential for harm.	‘I'm like a white female from a well-off background. So I don't know, is what I’m doing just tokenistic and ticking that EDI box, when actually, that's not what the purpose of it is. And I worry that I don't have the knowledge or the standpoint to be able to do something that's actually meaningful, like where I'm coming from I obviously don't have as good an understanding as someone who might be from a different background’. [P4, I1] ‘Being the straight white male, trying to include minority groups and represent them when I don't have that personal experience is something that I know myself, and a few other people, almost shy away from in case we end up accidentally stereotyping or creating the wrong impression. So, including diversity when we have not exactly a diverse team and individual is a bit of a challenge’. [P8, I1]
6. Knowledge of ethnic and social determinants of physical and mental health of **patients**	One participant (P3) contributed reflections on ethnic and social determinants of health and missed opportunities to incorporate this into simulation. Another (P5) described an educational context outside of simulation where the patient’s sociodemographic background impacted on the recommended management plan and the challenges that this presented to learners.	‘I think anything that brings health and social inequalities into the conversation in healthcare is so important because I think it's still severely lacking and it's often there in a kind of lighter token or elective module way, when the reality is they're the biggest determinants of the health of our patients’. [P3, I2] ‘The simulated patient comes from a lower socioeconomic background… the students trip up on, what recommendations they can give. Like they're saying eat healthy food and veg, do all these things and then the simulated patient will always come back with. “Well, I can't afford that. I work a full-time job. I can't do that.’ And it always kind of stumps the students because they don't know how to respond because most of our guidelines and things, there's like, ‘this is what a healthy diet looks like’. But it's not necessarily reflective of what certain parts of the population have access to’. [P5, I1]
7. Ability to reflect with students on the social or cultural context of the patient/**other professionals** relevant to the medical encounter	Participants spoke of not necessarily reflecting with students (or learners) directly in SBE but supporting them to reflect amongst themselves in the scenario debrief. They described this as an aspect of the role that they found difficult. Several (P2, P3, P4, P9) felt there was a tricky balance to strike between supporting reflection on EDI issues, whilst maintaining psychological safety within the learning space. Participants (P3, P9) spoke about simulation being a safe environment in which to support reflection on EDI issues, with the hope that this would lead to further personal reflection by the learners, which may then influence their clinical practice.	‘It’s a really complex group dynamic after sim because you really want to keep it as a safe space for the students… you really don't want them to have a negative experience. So how can you model behaviours or address concerning behaviours that might be reflected in sim, or that they might have raised that this made them think of outside the sim? How can you reflect that while keeping the psychological safety? I think is yes, the big challenge for me’. [P3, I1] ‘Issues will come up and if we don't feel equipped to manage them when they come up, then that can lead to loss of psychological safety. People not wanting to engage in sim again, people not getting the right learning out of the education that we're trying to achieve’. [P4, I1] ‘… sim is a good thing to do because that's where you just take that time to think through about your language and the way you've addressed people, the way you interact with people and how maybe you're not closed minded or narrow minded, but just in the heat of things, actually, the point is to learn in that safe environment so in the wide world, you don't make all these assumptions without thinking through how that might impact on somebody else's engagement with you’. [P9, I1]
8. Awareness that **simulation educators** are role models in the way they talk about **people** from different ethnic, cultural and social backgrounds, **professional roles and grades**	Role modelling was interpreted as being broader than the language and behaviours of individual educators. Participants considered the influence that educators can have through messages transmitted in the design and delivery of simulated scenarios. Participants (P1, P5, P6, P8) were aware that what is (or is not) said, or done, may signal their own, the team’s or the institution’s values and beliefs. One participant (P3) underlined how role modelling and messaging in EDI is present within simulation, regardless of whether a choice is made to consider and reflect on this within a programme. Several participants spoke of SBE as an environment essential for positive role modelling in EDI, recognising the potential for transfer to clinical practice and the impact of the hidden curriculum (P2, P5, P7).	‘What are we reflecting of our own judgements and perspectives in these sims because that's really what they are. We're creating them, so in our mind we picture that patient that's coming in with condition XYZ. So, I think we have to be very mindful of our own judgements coming through these’. [P1, I1] ‘I mean, that's the thing about EDI. It's incorporated either way. So, either way, we are reinforcing messages about race, gender, class. That's the thing about power structures, we’re reinforcing them either way. So, we either reflect on that and decide what messages we want to reinforce, or we ignore it and we continue to reinforce probably pretty negative messages’. [P3, I1] ‘Simulation, it’s an amplified kind of learning environment, so if we're modelling behaviours or seeing behaviours in that environment, you would hope there would be a leak out into the real world. And if you're seeing good behaviours modelled there, then you'd hope that people that are coming to our sessions will then take that out into the world with them, whether we're explicit about that's what we're doing or whether that's part of kind of the hidden curriculum, so to speak, of our sim programme… to me I think education feels like a place where you really can influence culture’. [P7, I1]
9. Empathy (understanding and compassion) for students of diverse ethnic, cultural, social **and professional** background	One participant (P1) spoke of the challenge in facilitating sessions for learners of whom they have no prior knowledge, and therefore no understanding of their background and needs. They highlighted that a lack of understanding of what learners feel when participating in simulation can lead to missed opportunities with respect to attempts to increase representation of different groups within our programmes. Another (P6) described feeling uncomfortable when observing a simulation session due to concern that an element of the debriefing was insensitive to the learner.	‘They come in, they sit down, they get a biscuit and we assume that everyone's comfortable, but actually I've got no idea really, the demographic, the background, the experiences, they can have such different experiences as FY1s’. [P1, I1] ‘One particular one would have been in a setting where someone, a senior consultant, was making an observation and I guess a useful clinical consideration about the overweight patient that they had, but in the room there was someone who would probably consider themselves overweight and they just made a kind of poorly placed joke about that, which I thought was going to be damaging to that person, just from a sort of compassionate point of view, that person might’ve thought, well, maybe I'm not really valued in this scenario or, just from my own understanding I think, well, that person then might fixate on that or worry about what people were thinking of them in that moment’. [P6, I1]
10. Ability to engage, motivate and let participate all students	One participant (P2) felt that considering EDI within simulation was necessary to create psychologically safe spaces where learners can contribute freely and openly. Another (P4) discussed the accessibility of simulation programmes and identified limitations for learners with visual or hearing impairments. One participant (P10) described the challenges of ensuring engagement, motivation, and participation of learners within simulation sessions for a group with mixed professional backgrounds. Another participant (P7) highlighted the ‘uncomfortableness’ of challenging EDI issues as they arise within sessions, whilst identifying this as something required to allow all learners to participate. Two participants (P1, P3) recognised that diversity is required within simulation for a diverse group of learners to feel engaged, motivated and able to participate. The question of whether this is being done in a way that is beyond tokenism was raised (P7).	‘If, coming back to that psychologically safe piece, you're going to have a psychologically safe learning space where participants all feel that they can speak openly, freely, without prejudice or judgement, it's almost implicit that the EDI issue should be incorporated, because you're never going to have a psychologically safe space where someone with a protected characteristic, or otherwise, feels discriminated against, or slightly prejudice against, whether that's because of how the facilitator is debriefing, or how the simulation is slightly phrased or what the other participants might say?’ [P2, I1] ‘I guess when I'm thinking about accessibility of locations and things like that, as per hospitals, locations are generally accessible, like lifts, and doors are wide enough and things if you were a wheelchair user, you could easily set up rooms slightly differently to make it accessible if someone had a disability. But I wonder about the impact of if you were visually impaired or hearing impaired, how that would be for simulation …’. [P4, I1] ‘I wonder if sometimes there can be a case, in multidisciplinary sims… where you can find that there are multiple disciplines in the sim, but the debriefs can sometimes be aimed at specific people rather than group, so I think you can have all these people involved, but not everyone benefits to the same extent’. [P10, I1]

Amendments to Hordijk et al.’s original competencies are presented in bold. *I1*, interview 1; *I2*, interview 2; *P*, participant number

### Inductively developed themes

Illustrative quotes for the following inductively developed themes can be found in Table 3.

**Table 3:** Illustrative quotes from interviews for inductively coded themes

Theme	Illustrative quotes
Team reflection on EDI	‘Because the people I'm doing the sim discussions with, with the sim faculty, are inherently a lot more reflective, and definitely more listening than I am, in terms of giving space to consider things, I think that is something I've more adopted. So that kind of openness to reflection and consideration as a group, rather than just kind of making up your own mind. I think is something that has changed for us… I think it's given people more confidence to talk about these things, kind of gives us permission to address them, which I think is nice as well. It's like I feel like maybe it's given a voice to other members of the faculty in general’. [P3, I2] ‘I think having sessions where we come together and can be open about issues that we have seen or where we have seen issues and we didn't feel that we maybe did as well as we could, and that it's OK to be quite open about that, and that we can have those discussions and you're not, you wouldn't be judged but it would just be a space where you can kind of share your inner landscape about what's going on’. [P7, I1] ‘I think particularly what comes up tends to be unconscious bias towards different specialties, and it's quite common, before we used the tool, just to kind of breeze past them, whereas actually it makes me think we should not be continuing to let unconscious biases go’. [P4, I2]
Collaboration	‘I would wonder, about involving our participants in these conversations at some stage too, because there's limits to my input and my awareness and I think I would learn quite a lot from the students or the participants because they'll be potentially better informed than I am on certain issues. They've obviously got different perspectives’. [P1, I2] ‘I think in general whether it's EDI or incorporating more empathy into our trainees, I think involving other groups in design and education could be highly beneficial. So, whether we're looking at something to do with power structure, homeless healthcare, talking to nursing staff for some of our communication stations, involving mixed multidisciplinary staff in our stations’. [P3, I1] ‘Well my feeling would be that you want to do it in partnership with either patient stories, or people who have been on the other end of it, rather than the people who are dramatising it thinking what it might be like, or thinking what these issues could be’. [P9, I1]

*I1* Interview 1, *I2* Interview 2, *P* Participant number

#### Theme 1: Team reflection on EDI

Participants highlighted the benefits of group reflection on EDI issues within simulation faculty. This was identified as an area worthy of development, in addition to personal reflection on values and beliefs (Competency 1) and reflection with students on social and cultural contexts (Competency 7). Participants spoke of reflective team conversations serving to highlight unconscious biases. One (P3) highlighted the process of group reflection as having given people more confidence to discuss EDI issues and ‘permission’ to address them openly. They also spoke of the process giving a voice to faculty members who may not otherwise contribute their ideas to programme development and how group reflection had positive impacts on personal reflection. Several (P2, P3, P4, P7, P10) spoke of the power of protecting time and space for team reflection in leading to action, for example through identifying and addressing unmet developmental needs of faculty or tackling EDI issues within programme design.

#### Theme 2: Collaboration

Participants discussed the importance of collaborating with others in the development of simulation programmes in order to ensure that changes made, and programmes developed, are informed by, and reflect the experiences of, the groups we are aiming to represent. Participants (P1, P3, P7, P8, P9) recognised the limits to their own awareness and understanding of EDI issues and the role that collaboration with patient groups, colleagues and learners, including co-creation of simulation programmes, plays in ensuring continued improvements within the education delivered.

### Construction of a modified competency framework in EDI for simulation educators

Through the analysis described above, and using data from interviews at both timepoints, we constructed a Developmental Framework for Simulation Educators in EDI (Table 4). Explanatory notes for the development of the framework can be found in Additional file 3. This framework differs from the original framework in that it is not focussed on competency to teach (or design) simulation specifically addressing EDI issues but rather as a basis of the competencies (or developmental areas) we feel are required to incorporate EDI into simulation programmes during the design, delivery and debriefing phases. We incorporated two additional themes of relevance to simulation faculty development in EDI which emerged inductively from the data (see above).

**Table 4:** Developmental framework for simulation educators in equity, diversity, and inclusion

Developmental areas	
1	Ability to critically reflect on own values and beliefs
2	Ability to communicate about individuals from ethnic, social, cultural **and professional** groups in a nondiscriminatory, non-stereotyping way
3	Empathy (understanding and compassion) for **all people, being mindful** of ethnicity, race or nationality, **sex, gender, cultural background, neurodiversity, socioeconomic status, body habitus**
4	Awareness of own ethnic and (sub)cultural background/standards **and those of the team delivering simulation education**
5	Knowledge of ethnic and social determinants of physical and mental health **and the impact of intersectionality (different interrelated dimensions of one person/patient, e.g. culture, social class, gender, disability, religion, sexual orientation)**
6	Ability to reflect with **learners** on the social or cultural context of the patient/**other professionals** relevant to the medical encounter **and with the simulation team on the design, content and delivery of simulation through an EDI lens**
7	Awareness that **simulation educators** are role models in the way they talk about **people** from different ethnic, cultural and social backgrounds, **professional roles and grades**
8	Empathy (understanding and compassion) for **learners** of diverse ethnic, cultural, social **and professional** background
9	Ability to engage, motivate and let participate all **learners**
10	**Ability to recognise the importance of collaboration and co-creation in the development of simulation education and to employ these practices wherever possible to enhance EDI within programmes**

Modifications and additions to the original framework are presented in bold text. See explanatory notes in Additional file 3 for further detail of how the original framework was modified and adapted based on the study data

## Discussion

In this study, we explored the relevance of a competency framework for medical teachers to simulation educators looking to incorporate EDI into their programmes. Through semi-structured interviews analysed using template analysis, we developed a new framework incorporating additional developmental areas identified from the data.

### Reflections on developing a modified framework in EDI

#### Intersectionality

Excerpts relating to 9 of the 10 competencies within the amended framework were present within our interview data, highlighting their relevance to the simulation context experienced by our participants. One competency, ‘Awareness of intersectionality’, had no excerpts coded to it. This does not, in the view of the researchers, mean that this competency is not relevant to simulation educators. On the contrary, we feel that an awareness of intersectionality is extremely important, and this finding may primarily represent a gap in awareness and knowledge amongst our participants. Hordijk et al. describe awareness of intersectionality as an ‘essential teaching competency’ in their discussion of the original framework [18]. Intersectionality describes the interaction between cultural and ethnic identity, gender, race and other categories of difference in people’s lives, social practices, institutional arrangements and cultural ideologies and the outcomes of these interactions in terms of power [18, 22]. The importance of integrating intersectionality into medicine and medical education has been presented by several academics [22–25] with reflexivity being identified as an integral process [24]. In our framework, we have incorporated intersectionality alongside knowledge of ethnic and social determinants of health. We feel this is appropriate as, in our experience, simulation educators are more often looking to consider EDI within their simulation programmes and debriefings, rather than necessarily deliver simulation addressing EDI-specific learning outcomes. We also incorporated reflection with colleagues into our framework. We would suggest that teams consider using the SIM-EDI tool [13], to promote and guide team reflexivity through structured conversation, as part of this reflective process.

#### Collaboration and co-creation

Two related themes that we incorporated into our framework are collaboration and co-creation within simulation education. Here, we recognise two important and interrelated issues. The first is the need for collaborative effort within simulation teams, and across institutions, to share innovations and best practice to promote educator development with respect to EDI. Simulation is inherently reliant on team delivery, and this, we feel, cannot be an individual endeavour. The second is the need for co-creation with minoritised and marginalised groups in all simulation education, not just those designed specifically for EDI learning outcomes. It is well established that the involvement of those with lived experience, and from the communities being represented within simulated scenarios, is imperative to authentic and non-tokenistic development of educational programmes [10, 26]. The addition of this to our developmental framework is important.

#### Translating increased awareness to sensitive facilitation

Increased awareness and recognition of EDI issues arising within simulation sessions was  evident within our participants over the course of the study period. The translation from increased awareness to the ability to address EDI issues when they arise remains a greater challenge. Ensuring a psychologically safe learning environment is maintained within sessions, through the appropriate and sensitive facilitation of discussions relating to EDI, is something that participants identified as an area where they lacked confidence. We propose that use of our framework to underpin faculty development in EDI will help support collaborative efforts within teams leading to identification of specific areas for development and local strategies to address these.

### Nomenclature

The original framework used as a basis for this study was Hordijk et al.’s framework for medical teachers’ competencies to teach ethnic and cultural diversity [18]. In line with current thinking in this field, we consider the framing of knowledge, understanding and skills relating to EDI in medical education as ‘competencies’ to be problematic [10, 13, 27–29]. Rather than being areas in which competence can achieved and then ‘ticked off’ in checklist fashion, we propose a shift to considering ‘developmental areas’, aligned more closely to the concept of cultural humility [30–32]. This approach recognises that progress can be made, but that ongoing self-awareness, openness and reflection are required [33], and that gaining confidence in EDI is an iterative process of lifelong development. A general feeling of low confidence and lack of expertise in EDI was a common concern amongst participants; this shift in nomenclature may also help to encourage those who feel under-skilled or lacking knowledge in this area to begin a journey of self-development

### Strengths and limitations

This is the first study that has proposed a developmental framework for simulation educators in EDI constructed through the exploration of faculty viewpoints using semi-structured interviews. Our framework incorporates the views and competencies that faculty ‘arrive with’ as well as those that they develop and/or become aware of during a period of enhanced consideration of EDI using a freely available reflexive tool, SIM-EDI. Therefore, our framework is designed to be relevant for simulation faculty at any stage of their career, including novice simulation educators. Though the number of participants is small, the use of individual semi-structured interviews allowed researchers to elicit rich narratives that informed the construction of our framework. Building on a pre-existing related framework, which had been developed in a Delphi study, provided a robust basis for our study.

The framework is the conceptualisation of the authors based on their interpretations of the interview data and their assimilation of the literature. The research team has some degree of diversity in ethnicity, gender, LGBTQ, religion and professional background. The interview data constitutes the perceived needs of faculty participants and cannot be interpreted as objective developmental needs. The study is limited by the small numbers of participants and the limited diversity within the participant group. All participants were working within the same simulation team and delivering the same simulation programme in one Health Board at the time of the study. A wider breadth of experience was shared in the interviews by some of the participants who have been involved in other simulation programmes. The input of members of the research team who work, or have worked, in other medical education settings (C. H. X. C., V. T., E. P., N. O.) has also, we hope, ensured broader relevance of the resultant framework.

### Areas for future work and research

This is the first iteration of a Developmental Framework for Simulation Educators in EDI. An area of potential focus henceforth is the development of guidance on how simulation educators and teams may address the developmental areas presented within the framework, outlining resources and educational activities of relevance to each area. A key avenue for future research lies in the use and study of the framework in other contexts, exploring its relevance and applicability. Exploration of how use of the framework influences faculty development, as well as subsequent impact on the development and delivery of simulation programmes, are other interesting areas for future research. Consideration should also be given to the incorporation of this framework into guidance and standards for simulation faculty development.

## Conclusion

Medical simulation educators must be equipped to address EDI in their simulation programmes. There is a recognised gap between acknowledgement of this and feeling empowered to act which has implications for faculty development. Here, we present work which has sought to close this gap through exploring the relevance to the simulation context of an existing competency framework and proposing a new Developmental Framework for Simulation Educators in EDI. We encourage simulation teams to utilise this framework within their faculty development programmes and report on their experiences.

### Supplementary Information

Additional file 1. Semi-structured interview schedule.

Additional file 2. Adaptations made to the original framework to form the amended framework.

Additional file 3. Explanatory notes for construction of the new developmental framework.

## Data Availability

The dataset generated is not publicly available to maintain participant confidentiality, but parts of the data are available from the corresponding author on reasonable request.

## Abbreviations

EDI: Equity, diversity and inclusion
JEDI: Justice, equity, diversity and inclusion
SBE: Simulation-based education
ASPiH: Association for Simulated Practice in Healthcare
SIM-EDI: Simulation equity, diversity and inclusion tool
NHS: National Health Service
