# Supplementary material for: Equity, diversity and inclusion in simulation-based education: constructing a developmental framework for medical educators
Source: Adv Simul (Lond). 2024 May 16;9:20. doi: 10.1186/s41077-024-00292-5 (PMC11097436; doi:10.1186/s41077-024-00292-5)
Supplement: Supplementary file 1 — Additional file 1. Semi-structured interview schedule. [file 41077_2024_292_MOESM1_ESM.docx]

**Semi Structured Interview Schedule, EDI in simulation, NHS Lothian**

| **Setup** | Notes/Progress |
| --- | --- |
| Thanks and introductions.  Interview will last around 30 minutes, be audio recorded and transcribed.  Once transcribed it will be held anonymously and only accessible to the research team.  If you would like to review your transcript please get in touch with lead researcher (JM) over the next two weeks.  The topic of discussion will be equity, diversity, and inclusion (EDI) in simulation.  Please feel free to skip over any questions that you do not wish to answer.  We can stop the interview at any point.  Check consent form received.  Are you happy to proceed?  *** Start recording (two devices)*** |  |
| **Introduction** |  |
| - Tell me a bit about yourself and your role on the simulation team - We’re talking about equity, diversity, and inclusion today - can you tell me about what those concepts mean to you? (1, 4, 5)^[[1]](#footnote-1)^ - Where do you think your understanding of these concepts has come from (1)?   - Explore formal training/in clinical training/personal reading etc. |  |
| **Current situation in NHS Lothian simulation** |  |
| - Can you describe your understanding of how/if equity, diversity, and inclusion is incorporated into simulation education in NHS Lothian currently?   - Design   - Delivery   - Debriefing - What are the potential impacts of the current setup in relation to EDI in simulation education in NHS Lothian? - Can you tell me about a time you were involved in a simulation session where issues related to equity/diversity/inclusion were relevant?   - Explore planned vs unplanned   - Explore types of themes that came up (e.g. race, class, power, gender, culture, sexuality) |  |
| **Challenging experiences** |  |
| - Have you ever been involved in a situation related to equity/diversity/inclusion in simulation that has made you feel uncomfortable?   - Can you describe it for me?   - What could have been done better?   - Do you have any thoughts on what would help in a similar situation in the future? - What do you find most challenging as a simulation facilitator when it comes to equity, diversity and inclusion?   - Why do you think that makes you uncomfortable/what makes that hard?   - Do you feel equipped to incorporate EDI into your simulation sessions?   - Do you feel able to deal with EDI issues as the arise? (1-8) |  |
| **Ideal** |  |
| - Do you think that issues relating to EDI should be incorporated into simulation education? - If yes, why do you think there is a need to incorporate EDI into simulation education (3, 6, 7)? - Do you think it is the role of the simulation educator to incorporate these issues? Why? - In an ideal world how do you think the simulation team would approach equity, diversity, and inclusion in simulation in NHS Lothian? - What is getting in the way of us doing that? - What do you think is needed to incorporate EDI into simulation education? - Is there anything else you would like to share with me today? |  |
| **Additional questions for second set of interviews** |  |
| **Experience with SIM-EDI tool** |  |
| - For the past few months the simulation team has been engaging in structured reflection after cases – tell me about that process. - Can you describe how those conversations impacted you as a simulation facilitator? - Were there any meaningful changes you noticed? - Do you feel there have been any impacts on your communication in relation to EDI issues? (2) - Has your own understanding of EDI changed over time? Can you explain why/how you think that has happened (1)? - Do you think there are potential benefits to clinical practice of incorporating EDI into simulation education? Can you say more about this? (3, 6, 7, 8) - How would you adapt this tool for future use? - If you were leading a simulation moving forward would you use the tool? Why/why not? - Do you feel equipped to incorporate the consideration of EDI issues into your simulation design, delivery and debriefing? (1-8) |  |
| **Future** |  |
| - What steps do you think the simulation service should take to enhance equity, diversity, and inclusion in simulation design, delivery and debriefing? - If you could change one thing in relation to EDI in simulation in NHS Lothian, what would it be? |  |
| **Additional comments** |  |
| - Is there anything else you would like to share today? |  |
| **Closing**  Thank you very much for taking the time to meet with me. I appreciate that this topic can be challenging to think about. Please let me know if there is anything that the research team can do to support you. |  |

Hordijk, R., Hendrickx, K., Lanting, K., MacFarlane, A., Muntinga, M. and Suurmond, J., 2019. Defining a framework for medical teachers’ competencies to teach ethnic and cultural diversity: Results of a European Delphi study. *Medical teacher*, *41*(1), pp.68-74.

Ten essential competencies for all medical teachers:

Execution of teaching

1. Ability to critically reflect on own values and beliefs
2. Ability to communicate about individuals from ethnic, social and cultural groups in a nondiscriminatory, nonstereotyping way
3. Empathy (understanding and compassion) for patients regardless of ethnicity, race or nationality
4. Awareness of intersectionality (different interrelated dimensions of one person/ patient, e.g. culture, social class, gender, disability, religion, sexual orientation)
5. Awareness of own ethnic and (sub)cultural background/standards
6. Knowledge of ethnic and social determinants of physical and mental health of migrants (e.g. risk factors, unfamiliar diseases, epidemiology (premigratory, migratory and postmigratory factors) and barriers to health care, relevant in the country, including undocumented patients
7. Ability to reflect with students on the social or cultural context of the patient relevant to the medical encounter (e.g. diagnose-telling and decision-making in case of cancer treatment, organ transplantation, palliative care)

Coaching of the learning process of students

1. Awareness that teachers are role models in the way they talk about patients from different ethnic, cultural and social backgrounds
2. Empathy (understanding and compassion) for students of diverse ethnic, cultural and social background
3. Ability to engage, motivate and let participate all students

1. Numbers in brackets correspond to competencies for medical teachers in Hordijk et al (2019) which are particularly relevant to the question – see below [↑](#footnote-ref-1)
