# Supplementary material for: Equity, diversity and inclusion in simulation-based education: constructing a developmental framework for medical educators
Source: Adv Simul (Lond). 2024 May 16;9:20. doi: 10.1186/s41077-024-00292-5 (PMC11097436; doi:10.1186/s41077-024-00292-5)
Supplement: Supplementary file 2 — Additional file 2. Adaptations made to the original framework to form the amended framework. [file 41077_2024_292_MOESM2_ESM.docx]

Additional File 2: Adaptations made to the original framework to form the amended framework

Adaptations in bold.

| **Original framework competencies from Hordijk *et al’s* framework for medical teachers’ competencies to teach ethnic and cultural diversity** | **Amended framework competencies, where different to the original framework (amendments in bold)** | **Justification for, and timing of, changes made (before or during analysis)** |
| --- | --- | --- |
| Execution of teaching | | |
| 1. Ability to critically reflect on own values and beliefs |  |  |
| 1. Ability to communicate about individuals from ethnic, social and cultural groups in a non-discriminatory, non-stereotyping way | Ability to communicate about individuals from ethnic, social, cultural **and professional** groups in a non-discriminatory, non-stereotyping way | Broadened during analysis to include professional groups. Interprofessional issues were commonly arising within the interviews in respect to healthcare SBE. |
| 1. Empathy (understanding and compassion) for patients regardless of ethnicity, race or nationality | Empathy (understanding and compassion) for **people,** regardless of ethnicity, race or nationality, **sex, gender, cultural background, neurodiversity, socioeconomic status, body habitus** | Broadened before analysis to encompass the range of differences encountered and arising within simulation education and to acknowledge the importance of empathy for those other than patients (e.g. colleagues, relatives) within the healthcare setting. |
| 1. Awareness of intersectionality (different interrelated dimensions of one person/patient e.g. culture, social class, gender, disability, religion, sexual orientation) |  |  |
| 1. Awareness of own ethnic and (sub)cultural background/standards | Awareness of own ethnic and (sub)cultural background/standards **and those of the team delivering simulation education** | Positionality of the wider team incorporated during analysis as being discussed by faculty within interviews. Acknowledges the team-based design and delivery of simulation education. |
| 1. Knowledge of ethnic and social determinants of physical and mental health of migrants (e.g. risk factors, unfamiliar diseases, epidemiology (premigratory, migratory and postmigratory factors) and barriers to health care, relevant in the country, including undocumented patients (75%) | Knowledge of ethnic and social determinants of physical and mental health of **patients.** | This competency originally related specifically to migrant health. We broadened the definition, prior to analysis, to widen the applicability of the framework. |
| 1. Ability to reflect with students on the social or cultural context of the patient relevant to the medical encounter (e.g. diagnose-telling and decision-making in case of cancer treatment, organ transplantation, palliative care) | Ability to reflect with students on the social or cultural context of the patient/**other professionals** relevant to the medical encounter | Competency broadened during analysis to reflect interprofessional interactions within simulation-based education, the impact of EDI on these and the potential resultant impact on the medical encounter, recognising this as a commonly discussed issue within the interviews. |
| Coaching of the learning process of students | | |
| 1. Awareness that teachers are role models in the way they talk about patients from different ethnic, cultural and social backgrounds | Awareness that **simulation educators** are role models in the way they talk about **people** from different ethnic, cultural and social backgrounds, **professional roles and grades** | Competency modified before analysis to specifically identify simulation educators. Broadened during analysis to capture power differentials and hierarchy, as relevant to medical simulation, and broadened to include those other than patients (colleagues, relatives) noting this as being relevant in healthcare simulation. |
| 1. Empathy (understanding and compassion) for students of diverse ethnic, cultural and social background | Empathy (understanding and compassion) for students of diverse ethnic, cultural, social **and professional** background | Competency broadened during analysis, due to content of interviews, to include professional background of learners given that simulation may be delivered for people from varying professional backgrounds which may influence their interpretation of, and engagement with, the material, |
| 1. Ability to engage, motivate and let participate all students |  |  |

**Explanatory notes for adaptations made to the original framework prior to and during analysis.** In general, adaptations were made in order to align the framework with our conceptualisation of equity, diversity and inclusion (EDI) within the study, broadening this from ethnic and cultural diversity only, and to increase relevance for simulation-based medical education. Changes were made through discussion between researchers JM and SG prior to coding, and further modified during the coding process as the data were better understood.
