# Supplementary material for: Equity, diversity and inclusion in simulation-based education: constructing a developmental framework for medical educators
Source: Adv Simul (Lond). 2024 May 16;9:20. doi: 10.1186/s41077-024-00292-5 (PMC11097436; doi:10.1186/s41077-024-00292-5)
Supplement: Supplementary file 3 — Additional file 3. Explanatory notes for construction of the new developmental framework. [file 41077_2024_292_MOESM3_ESM.docx]

Additional File 3: Explanatory notes for the construction of the new developmental framework

| **Developmental Area** | | **Notes on amendments made to the original framework and modifications arising from the data** |
| --- | --- | --- |
| 1 | Ability to critically reflect on own values and beliefs | No amendment made. |
| 2 | Ability to communicate about individuals from ethnic, social, cultural **and professional** groups in a non-discriminatory, non-stereotyping way | Amendments as per Additional File 2. |
| 3 | Empathy (understanding and compassion) for **all people,** **being mindful** of ethnicity, race or nationality, **sex, gender, cultural background, neurodiversity, socioeconomic status, body habitus.** | Amendments as per Additional File 2. In addition, ‘regardless of’ was changed to ‘being mindful of’ to reflect our conceptualisation that empathy requires deliberate consideration of a person’s background, as well as their experiences in relation to their values and beliefs. |
| 4 | Awareness of own ethnic and (sub)cultural background/standards **and those of the team delivering simulation education** | It became clear through the interview data, and through our own review of the literature, that positionality, both on an individual and team level was important to the endeavour of healthcare SBE. |
| 5 | Knowledge of ethnic and social determinants of physical and mental health of **and the impact of intersectionality (different interrelated dimensions of one person/patient e.g. culture, social class, gender, disability, religion, sexual orientation)** | This developmental area incorporates intersectionality (Competency 4 in the original framework) as we consider these to be inter-related concepts. |
| 6 | Ability to reflect with **learners** on the social or cultural context of the patient/**other professionals** relevant to the medical encounter, **and with the simulation team on the design, content and delivery of simulation through an EDI lens** | Students changed to learners to better reflect the varied participants in simulation. Team reflection, an inductively coded theme, is incorporated here alongside reflection with learners. Broadening to include professionals during analysis as per Additional File 2. |
| 7 | Awareness that **simulation educators** are role models in the way they talk about **people** from different ethnic, cultural and social backgrounds, **professional roles and grades** | Modified before and during analysis as per Additional File 2. |
| 8 | Empathy (understanding and compassion) for **learners** of diverse ethnic, cultural, social **and professional** background | Students changed to learners to better reflect the varied participants in simulation. Broadened during analysis as per Additional File 2. |
| 9 | Ability to engage, motivate and let participate all **learners** | Students changed to learners to better reflect the varied participants in simulation. |
| 10 | **Ability to recognise the importance of collaboration and co-creation in the development of simulation education and to employ these practices wherever possible to enhance EDI within programmes** | Developmental area added to incorporate the inductively coded theme of collaboration. |

*Modifications in bold.*
